# The impact of spoken action words on performance in a cross-modal oddball task

**DOI:** 10.1371/journal.pone.0207852

**Published:** 2018-11-20

**Authors:** Gregory Neely, Daniel Eriksson Sörman, Jessica K. Ljungberg

**Affiliations:** 1 Department of Psychology, Umeå University, Umeå, Sweden; 2 School of Psychology, Cardiff University, Cardiff, United Kingdom; University of Amsterdam, NETHERLANDS

## Abstract

In this study a cross-modal oddball task was employed to study the effect that words spoken either non-urgently or urgently would have on a digit categorization task and if women would exhibit greater behavioral inhibitory control. The words were unrelated to the task itself, but related to the action required to complete the task. Forty participants (21 women) conducted a computerized categorization task while exposed to a sinewave tone as a standard stimulus (75% of the trials) or a to-be ignored word (*press*, *stop*) spoken either non-urgently or urgently as unexpected auditory deviant stimulus (6.25% trials for each category). Urgent words had sharp intonation and an average fundamental frequency (F_0_) ranging from 191.9 (*stop*) to 204.6 (*press*) Hz. Non-urgent words had low intonation with average F_0_ ranging from 103.9.9 (*stop*) to 120.3 (*press*) Hz. As expected, deviant distraction and longer response times were found by exposure to the word *stop*, but deviant distraction was not found to be significant with the word *press* or due to intonation. While the results showed that women had in general longer reaction times, there were no gender differences found related to the deviant distraction caused by word or intonation. The present results do not support the hypothesis that women have greater behavioral inhibitory control, but there was evidence that the meaning of the word could influence response times.

## Introduction

The detection of unexpected auditory events is of paramount importance because it can signal changes or dangers in the environment. The sentinel quality of the auditory system stems partly from its diurnal accessibility (ears are never shut) but also from its capacity to automatically register and attend to novel or deviating auditory events [1, 2]. A well-established method to experimentally measure such events is the oddball task that originates from electro-physiological work demonstrating brain responses specific to the detection of unexpected events and the automatic orienting of attention to them [3–5]. The general paradigm for this method entails conducting a primary task directly after the presentation of to-be-ignored stimuli, where the stimuli are composed of a high probability standard stimulus which is randomly exchanged with unique (novel) or infrequent, but repeated (deviant) “odd” stimuli. Typically in this paradigm three specific brain patterns have been identified which are associated with the involuntary distraction caused by novel or deviant stimuli: mismatch negativity (MMN), P3a and reorientation negativity (RON). The MMN response reflects an automatic change detection brought on by discriminable change in an otherwise repetitive auditory stream [6]. The P3a, or novelty-P3, marks the automatic orientation towards the detected change [5, 7] while the RON response is present when attention is directed back to the primary task [4, 8, 9].

Importantly, deviating sounds also have a clear behavioral impact. Studies using this type of task across two modalities (cross-modal oddball task) have shown that rare and unexpected sounds can involuntary orient attention away from a task at hand and delay responses in that task [10–13]. Most commonly, participants perform a computerized visual task while being exposed to repeated auditory sounds. During most of the trials the same sound is repeated (standard) and on rare and unpredictable occasions, a new sound is presented. The new sound is labelled a deviant when it is an unexpected change from the standard sound but recurs periodically during the experiment and is labelled a novel if each new sound is unexpectedly presented and is unique in nature. Both novel and deviating sounds can activate an involuntary switch of attention and create distraction in the focal task, but to a different degree [10]. Through this methodology, the attentional and orienting effects between distractor and primary task that have been observed in electrophysiological data have been replicated. Deviance distraction has also been found to be mediated by factors such as age, emotion, memory load, and cognitive control (see [14] for a review).

Spoken words are sometimes used as to-be ignored deviants in cross-modal oddball studies to capture attention away from a primary task [11, 13, 15]. Interestingly, Ljungberg et al [13] showed that deviating spoken words with a sharp intonation (urgently spoken) compared to non-urgently spoken words can involuntary speed up a response in a cross-modal oddball task. Arrabito et al [16] found a similar result investigating effects of verbal cockpit warnings on performance of a visual pursuit tracking task.

A few studies have found indications that action related deviant words result in a deeper semantic processing. For example, brain imaging studies have shown that, following the onset of a spoken word with action content embedded in sentences, cortical activity outside the typical language areas has been detected in regions that are related to motoric control [17–19]. Induced by the action words, somatotopic activation of the sensorimotor cortex has also shown differentiation in brain activity depending if a spoken or written word is related to leg, arm or face movements (see [20] for a review). Shtyrov et al [21] used an oddball paradigm with spoken words (*kick*, *pick*) as deviants demonstrating that a hand related action word (pick) elicited a widespread lateral distribution while a foot related word (kick) activated a more focal dorsal negativity. Furthermore, activation is rapid (150–200 ms) and thought to be independent of focused attention [22].

As far as we know, no study has studied the behavioral relationship between the semantic content of the to-be ignored distractor word and gender. Nevertheless, there are indications that gender is a mediating factor of the processing differences and behavioral responses to spoken words. For example, in related research paradigms, studies have shown that women have a more automated semantic processing (i.e. larger and earlier N400 effect) of spoken words [23] as well as of visual materials using word-pairs [24]. Women seem to process the emotional prosody in spoken words earlier and show both behavioral and ERP priming effects to a larger degree than men when the intervals between prime and target are shorter [25]. Furthermore, women more than men integrate emotional cues from the intonation of spoken words, which affect the processing of the semantic content [26]. Similar conclusions have been drawn from results in visual priming studies [27, 28]. It is also commonly agreed that the language processing in men and women differ because of the different organization of the spoken language in the brain. Men have a stronger left hemisphere lateralization while women seem to process language more bilaterally [29, 30] and this might explain why women process verbal material more efficiently [31]. In addition to this, women tend to show less deviant distraction in modified versions of the oddball task in which visual stimuli have been used, and wherein participants categorized pictures as standard and novel (see e.g. [32, 33]). Gender differences were, however, not found for response times in the standard trials. Thus, results suggest that women can make use of greater behavioral and inhibitory control compared to men in oddball settings. Based on previous findings on processing differences between women and men, both with regard to word processing and sensitivity to distraction, it seems likely that women would outperform men in oddball tasks that include also semantic deviants.

The overall aims of this study were to investigate differential effects of words spoken either non-urgently or urgently on deviant distraction, and if women would exhibit greater behavioral inhibitory control (i.e. less deviant distraction) than men. We designed an experiment to investigate deviance distraction in a cross-modal oddball task where the deviants were spoken action words which vary in intonation in order to bear information on urgency. The deviant words, *stop* and *press*, were related to the action required to respond to the primary task, classification of odd and even numbers presented visually, but unrelated to the actual task. It was expected that: 1) Deviance distraction will be found for all four deviant conditions (*Stop* Non-urgent deviants, *Stop* urgent deviants, *Press* Non-urgent deviants, *Press* Urgent deviants) compared to the standard (a tone). 2) Urgently spoken words with sharp intonation were expected to shorten response times compared to non-urgent words with low intonation, independent of the type of word. 3) Women were expected to process the content of the spoken word deviants faster than men and inhibit an involuntary response to speed up when exposed to the deviant *press* prior to a visual target and inhibit an involuntary response to withhold when exposed to the deviant *stop* prior to a visual target.

## Methods

### Participants

Forty (21 women) participants took part in this experiment in exchange for a small honorarium. Participants age ranged between 20 and 38 years (women: *M* = 24.4, *SD* = 2.9, men: *M* = 25.7, *SD* = 5.3), all were native Swedish speakers and reported normal or corrected-to-normal vision and normal hearing.

### Stimuli, design and procedure

**Fig 1:**
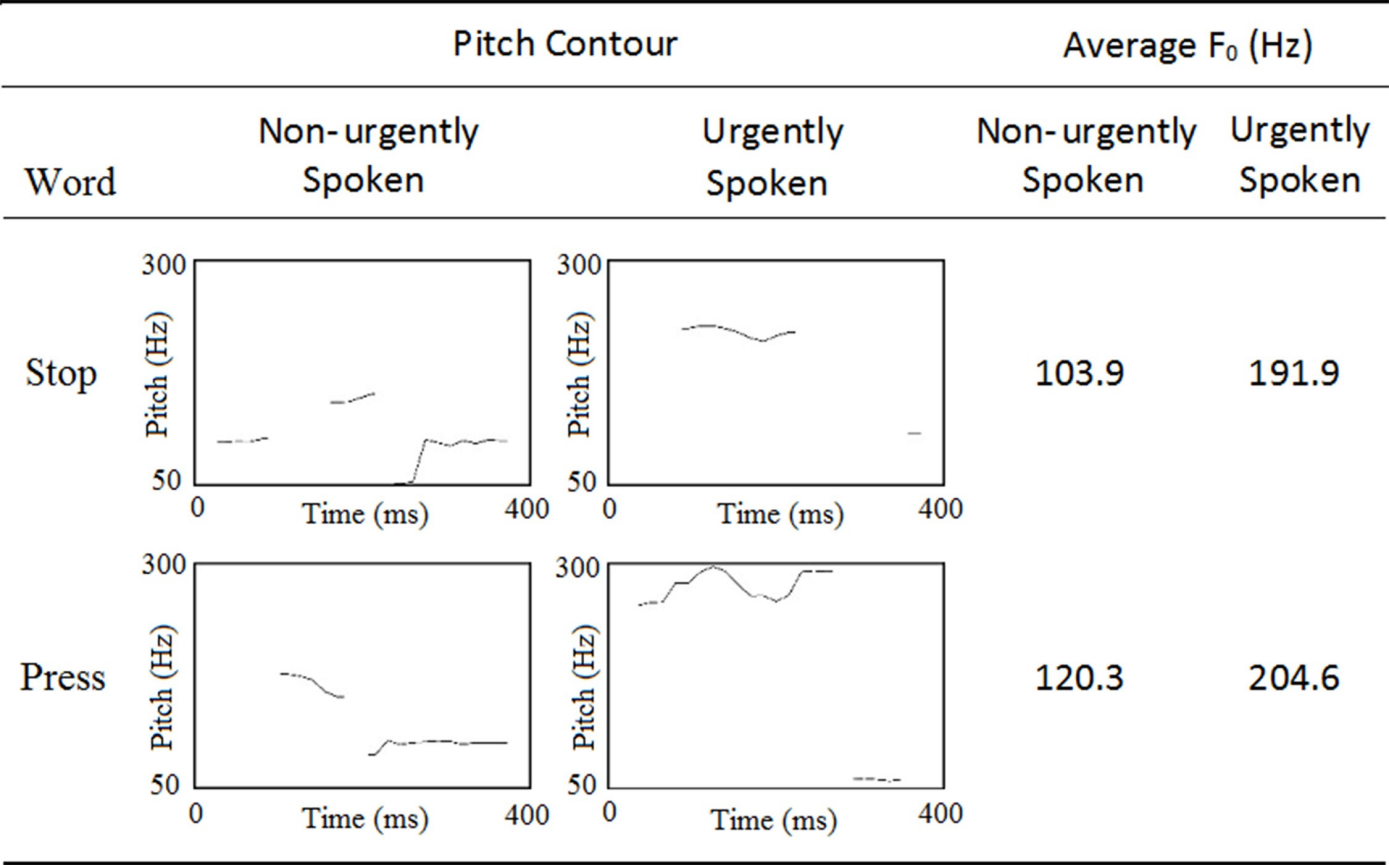
Pitch contour and average fundamental frequency (F_0_) for all words as a function of spoken style.

Five auditory stimuli were used. One standard sound consisting of a sinewave tone (600 Hz) and the words *stop* and *press* (in Swedish: *stopp*, *tryck*) spoken either in an urgent or non-urgent way were used as deviants. All words were recorded in the same male voice and sampled with a resolution of 16-bit and a sampling rate of 44.1 KHz by using Audacity 1.3 beta software. The word deviants and the standard sound were normalized, and digitally edited so as to be 400 ms in length with 10 ms rise and fall ramps. Following the method used by Edworthy et al [34], urgently spoken words were created by instructing the speaker to utter the words as he would to warn a loved one of a potentially dangerous situation. For the non-urgent words he was instructed to speak as if there was nothing special about the words. Pitch contour and average fundamental frequency (F_0_) for all words as a function of spoken style are presented in Fig 1.

The cross-modal oddball task (see description below) was programmed in E-Prime 2.0 [35] and executed on a PC computer using Microsoft Windows 7 Enterprise with a 24-inch widescreen LCD-monitor. All the sounds were presented to the participants binaurally through headphones (Vic Firth) at a sound level between 70-74dB(A).

The cross-modal oddball task consisted of 4 blocks of 384 test trials; including 288 standard trials (75%), 24 *stop* non-urgent deviants (6.25%), 24 *stop* urgent deviants (6.25%), 24 *press* non-urgent deviants (6.25%) and 24 *press* urgent deviants (6.25%). Each block of test trials was preceded by 12 practice trials consisting of standard trials. Each trial started with the presentation of a 400 ms auditory distractor (standard or deviant) that participants were instructed to ignore. With a stimulus onset asynchrony (SOA) of 100 ms, a digit (1–6) appeared at the center of the screen for 300 ms, which participants had to categorize as odd or even by pressing one of two response keys (Z or X, the mapping of response category to keys was counterbalanced across participants). A white fixation cross was visible at the center of the black screen throughout the task except during the presentation of that digit (presented in white as well) and subtending a viewing angle of approximately 2.6°. The digits were randomly presented (in a different order for each participant) but with equal probabilities of occurrence across each of the conditions (standard, *stop* non-urgent, *stop* urgent, *press* non-urgent, *press* urgent). Following the disappearance of the digit, participants had an additional 700 ms to respond before the next trial automatically started. The deviant words were randomly dispersed across trials with the exception that they were separated by at least one standard trial.

Prior to the experiment, the participants received oral and written instructions about the task and the procedure. All participants were instructed to respond as quickly and accurate as possible, to keep the headphones on during the whole experiment and to ignore all sounds. The experiment lasted approximately 40 min.

### Ethical considerations

Individual informed written consent was obtained before the start of the study. The participants were assured that they could leave the study whenever they wished to. The study was approved by the Regional Ethical Review Board in Umeå (2012-337-31Ö).

### Statistical analysis

Given the finding that the first standard trial following a deviant trial yields some residual distraction [36, 37], these trials were excluded from all analyses. Both response accuracy and mean response times were examined. Mixed ANOVAs with repeated measures on condition (standard, stop urgent, stop non-urgent, press urgent, press non-urgent) and gender as a between-groups variable were carried out on both accuracy and response time to determine if the presentation of word deviants yielded distraction relative to the standard condition. Where sphericity could not be assumed, Greenhouse-Geisser corrections were made. Next, deviance distraction was calculated by subtracting participants mean response time in the standard trails from the mean response times in the conditions where a deviant was present. Mixed ANOVAs with repeated measures on these variables were then examined to further explore the effects of deviant distraction with gender as between groups variable. Bayesian analysis were conducted using the JASP software [38] on the data in order to help interpret findings.

## Results

### Accuracy

Overall, participants performed well. Mean accuracy (proportion of correct responses) in the five conditions varied between .90 and .91 (standard deviations between .06 and .08). A mixed ANOVA was carried out on the accuracy with repeated measures on condition (standard, *stop* urgent, *stop* non-urgent, *press* urgent, *press* non-urgent) and Gender (male, female) as a between group variable. No main effect of condition (*F*(4, 152) = 1.58, *MSE* = .001, *p* = .182, $\eta_{p}^{2}$ = .04, Cohen’s *d* = 0.41) nor gender (*F*(1, 38) = .522, *MSE* = .012, *p* = .474, $\eta_{p}^{2}$ = .014, Cohen’s *d* = 0.24) was found; neither was there any interaction effect (*F*(4, 152) = 1.19, *MSE* = .001, *p* = .318, $\eta_{p}^{2}$ = .03, Cohen’s *d* = 0.35).

### Response times

**Fig 2:**
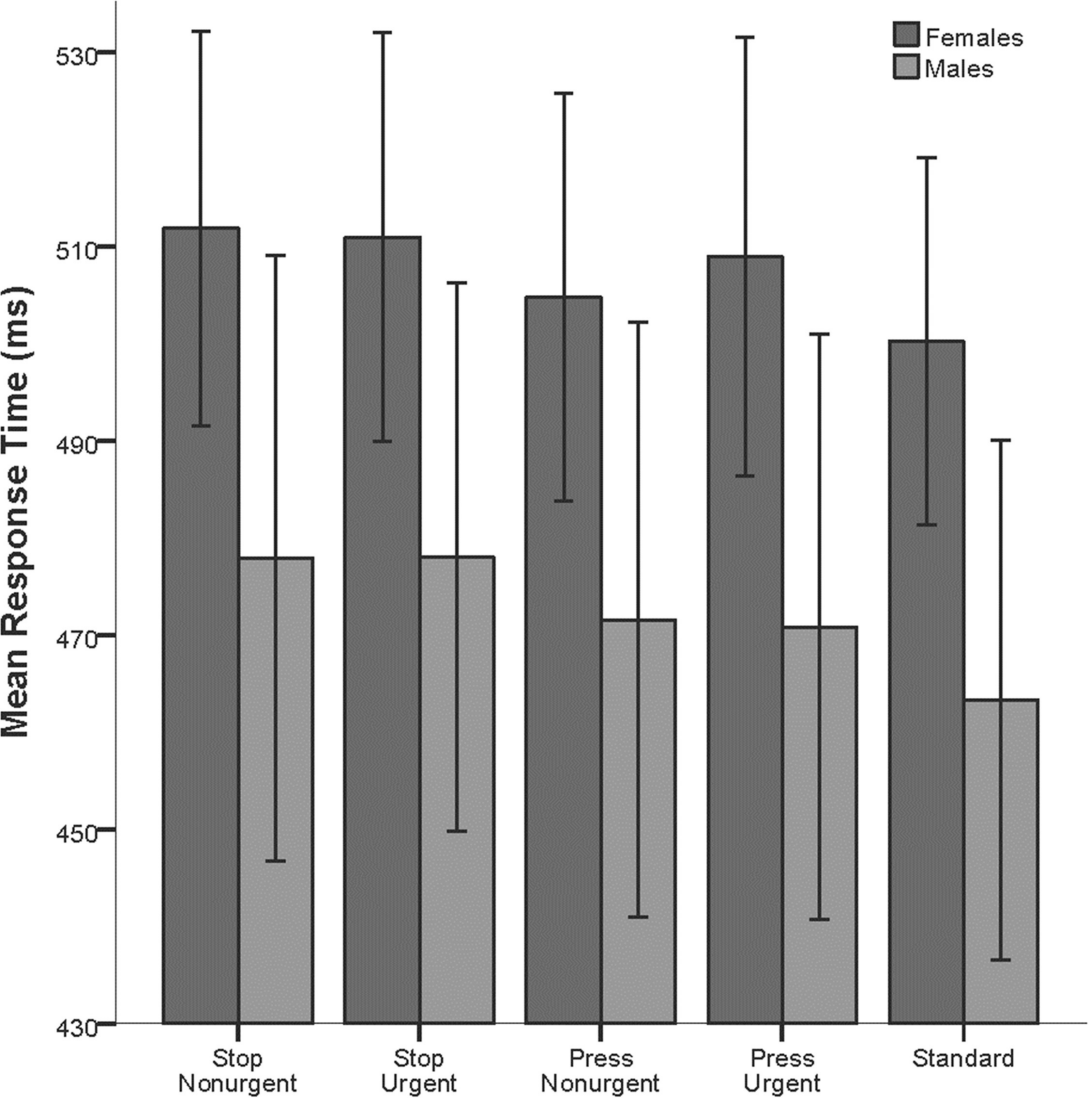
Mean response times (ms) and 95% confidence intervals for each condition for females (n = 21) and males (n = 19).

Average response times for males and females in the five conditions are presented in Table 1 and illustrated in Fig 2. A mixed ANOVA was carried out on the response times with repeated measures on Condition (standard, *stop* urgent, *stop* non-urgent, *press* urgent, *press* non-urgent) and Gender (male, female) as a between group variable. Significant main effects were revealed for condition (*F*(2.88, 109.83) = 6.11, *MSE* = 1594.84.2, *p* = .001, $\eta_{p}^{2}$ = .139, Cohen’s *d* = 0.80) and gender (*F*(1, 38) = 4.47, *MSE* = 61158.76, *p* = .041, $\eta_{p}^{2}$ = .105, Cohen’s *d* = 0.69); however, there was no interaction effect (*F*(4, 152) = .288, *MSE* = 54.17, *p* = .885, $\eta_{p}^{2}$ = .008, Cohen’s *d* = 0.18). Thus, female response times were longer than male response times for all conditions, and this difference between genders was similar for all conditions. Bayesian analysis revealed that there was strong evidence in favor of a model containing only the main effects over the model that included the interaction (BF = 0.05). With regard to condition, pairwise comparison using a Bonferroni adjustment for multiple comparisons revealed significantly slower response times for the two conditions when the word *stop* was used (*p* < .001 when spoken non-urgently and *p* < .01 when spoken urgently), but not for conditions when the word *press* was used (*p* = .99 when spoken non-urgently and *p* = .45 when spoken urgently) compared to standard trials. There was only anecdotal evidence for or against the null hypothesis when comparing the two *press* conditions to the standard trials (BF_10_ = 0.56 when spoken non-urgently and BF_10_ = 1.26 when spoken urgently); however there was extreme or very strong evidence that reactions times where slower than the standard trails in the *stop* conditions (BF_10_ = 265.74 when spoken non-urgently and BF_10_ = 32.61 when spoken urgently).

**Table 1:** Mean response times (ms) and standard deviations for each condition for the total sample, females, and males.

	Total Sample (n = 40)		Females (n = 21)		Males (n = 19)	
Condition	Mean	SD	Mean	SD	Mean	SD
Standard	482,67	51,68	500,21	41,51	463,28	55,83
*Stop* Non-urgent deviants	495,74	57,14	511,86	44,61	477,91	65,00
*Stop* Urgent deviants	495,29	54,58	510,94	46,20	477,99	58,99
*Press* Non-urgent deviants	488,99	57,09	504,76	46,14	471,57	63,92
*Press* Urgent deviants	490,84	58,78	508,92	49,62	470,85	62,83

**Fig 3:**
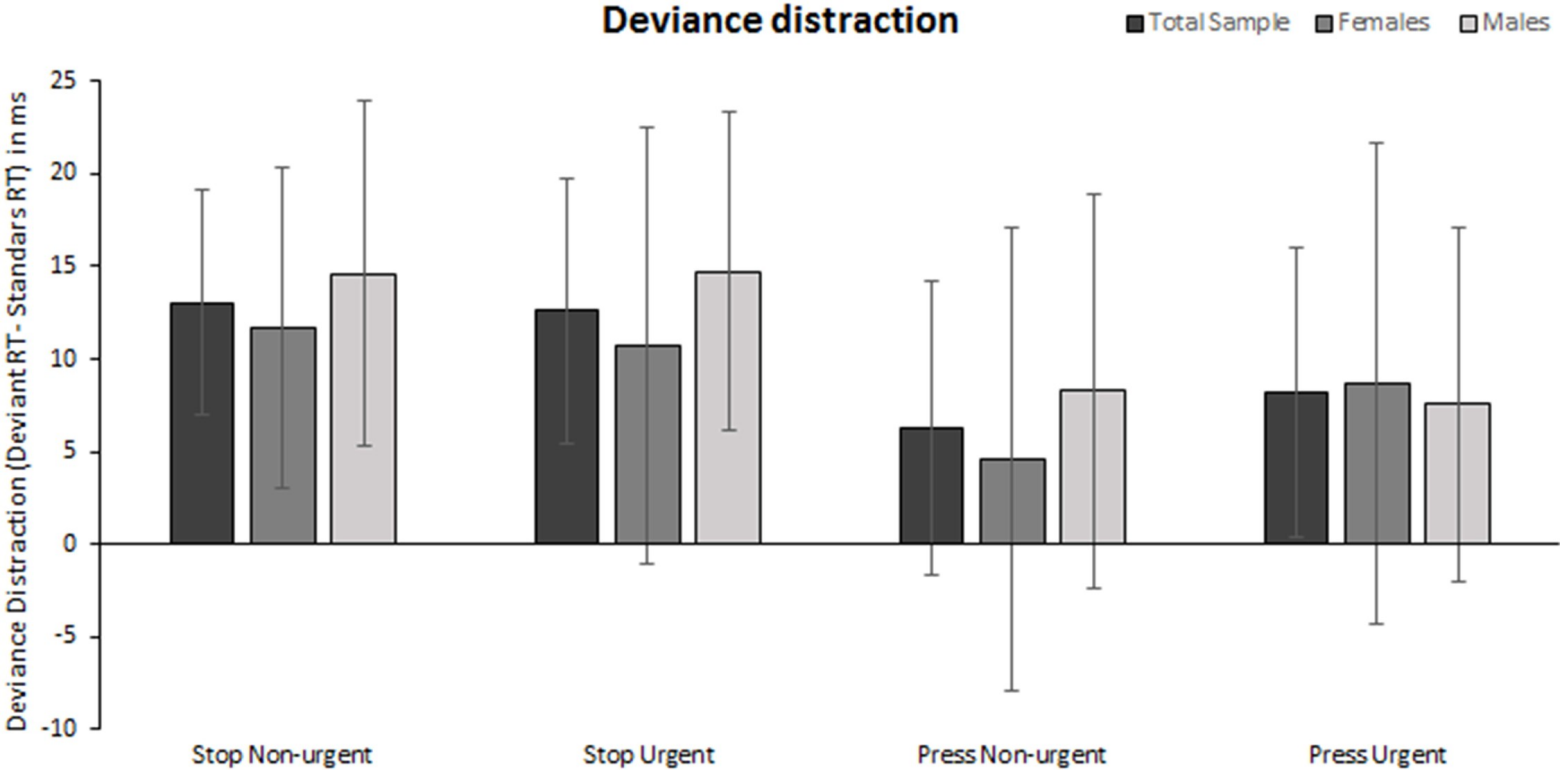
Average deviance distraction (ms) and 95% confidence intervals for females (n = 21), males (n = 19), and the total sample (n = 40) in each condition.

To further investigate the impact of deviance distraction, a two-way mixed ANOVA on intonation (urgent, non-urgent) and word (stop, press) with gender as between groups variable was conducted using the variables calculated as the difference in response times in deviants compared to the standard trials. The results are presented in Fig 3. A significant main effect was found only for word (F(1, 38) = 8.04, MSE = 1272.3, *p* = .007, $\eta_{p}^{2}$ = .175, Cohen’s *d* = 0.92). Distraction was greater when stop was present. No other effects were significant (F < 1, *p* >.432 in all cases). Bayesian analyses indicated that the model containing the main effect of intonation was more than 5 times less likely than the null hypothesis model (BF_01_ = 5.58) while the model containing the main effect of gender was marginally more than two times less likely than the null hypothesis model (BF_01_ = 2.15). There was moderate evidence (BF_10_ = 5.58) in favor of the model containing only the main effect of word over the model containing the null hypothesis. That no main effect of gender was present could be expected considering that overall gender differences in response times has been taken into account calculating difference scores. As anticipated based on the previous analyses there was no interaction effect between intonation or word and gender. Bayesian analyses revealed that there was moderate evidence in favor of models containing only the main effects when looking at intonation and gender (BF = 4.14) and word and gender (BF = 4.13) over the models that included the interaction.

## Discussion

Deviance distraction was statistically significant only when the word *stop* was present. Parmentier et al [15] have shown that semantic deviants that are congruent to the target of the primary task (e.g. the word *left* spoken pre-target when the target stimuli to categorized is an arrow pointing left) facilitate response times enough to cancel out deviance distraction. While the semantic deviants used in the present study were not directly related to the targets, they were related to the action that was needed to be performed during the response. Thus, as was seen in the case of congruent distractor-target, it is likely that deviance distraction was counteracted by the facilitating effect that the congruent action word (press) had on the response mode. However, it should be noted that the Bayesian analysis showed the data provided weak support in favor of slower reaction times when press was spoken urgently. Although a facilitating effect of the word *press* may have cancelled out or diminished deviant distraction, it should still be stressed that effects of distraction were found in the conditions where the word *stop* was used which effectively delayed response times. This is an interesting finding, and in accordance with expectations. Thus, this study can confirm that words unrelated to the task itself, although related to the actions required, can cause deviant distraction, but that such effects seem to be influenced by the semantic meaning of the deviant and the action to be performed.

It was expected that an urgent intonation would quicken response times, however no evidence for this was found. In a previous study [13] an urgent intonation to words facilitated quicker response times (decreased deviant distraction) compared to the same words spoken without urgency. There are, however, two key differences between the present study and this previous work. First, the semantic deviants in the previous study were not related in any way to the primary task. Thus, it could be that the informational value of the semantic deviants used in the present study trumped any additional information that intonation may have brought. Second, the deviants in the previous study were comprised of 8 words as opposed to only two in the present study. Given the more limited variation and the greater repetition of the semantic deviants in the present study, there could have been a habituation effect.

While there was an overall gender effect, with women responding significantly slower than males, there was no indication that women performed differentially in respect to action word or intonation. Previous studies have indicated that women can assert greater behavioral control, which we anticipated would modulate the response times in accordance to whether the semantic deviant was congruent or incongruent with the action to be performed.

While modified visual oddball studies [32, 33] indicate that women show less distraction than men, they generally do not find any difference in response times in standard trials. Both of these findings are in direct conflict with the current results. Part of the discrepancy may be due to the fact that Yuan et al [32, 33] used novel distractors and the task to be completed was categorizing the stimuli as either the standard picture or a novel picture. No to-be-ignored stimuli were used and only visual stimuli were used. Jaušovec and Jaušovec [39] have shown that females’ visual event-categorization process is more efficient than males in a task that did not include any rare or deviant stimuli, so it would appear that adding a novel distraction does change the dynamics to a visual categorization task. Using an auditory/visual cross-modal oddball design with novel auditory distractors, Garcia-Garcia et al [40] found a significant interaction effect between emotional context and novelty for females where the distraction effect on response time was significantly larger in the emotionally-negative context, while no interaction effects were found for males. However, in their study men’s and women’s behavioral results were never analyzed as a between groups factor and thus it is difficult to directly compare response times. In the present study, gender did not modulate deviance distraction and it may be that emotional context is necessary to create a gender difference.

Gender differences in simple response time (SRT) and choice response time (CRT) have been extensively studied [41–43] with the consistent finding that in the SRT condition men respond faster. The results with CRT has been less clear with some studies finding men respond faster [42, 44] while others finding no differences [43, 45]. In the latter case, the lack of a difference has been attributed to faster decision times counteracting slower movement times in the female’s performance compared to the male’s performance. However, as Dykiert et al [43] point out, contradictory results have been achieved using the exact same paradigm and exact same equipment. The task employed in the present study is a CRT task; however, the previously cited studies using CRT tasks have employed far more choice alternatives (4 or more) than the current study (only 2 alternatives) and thus in a continuum of complexity fall closer to a SRT task where gender differences are consistently found than the CRT tasks employed in studies finding no gender differences.

The results from the present study indicate that urgency of spoken words might be less important than meaning of the words, at least in situations where the words are related to the task at hand and not in competition with a large set of distractors. We found that the word *stop* effectively delayed response times in a visual task (where the word was related to the action to be performed to complete the task, but not the task itself) while the word *press* did not affect response times. Deviants in a cross-modal oddball typically result in longer response times to the primary task. That response times were not affected by the word *press* may reflect a facilitation of the expected response times caused by action associated with the word. While women overall had slower response times, their responses were not differentially affected by the word or intonation manipulations. As the present study’s methodology differs in many key ways to previous studies of gender differences to sensory distraction caused by auditory semantic stimuli, the current results do not conclusively reject the possibility that women have greater behavioral inhibitory control as well as more efficient processing of semantic content. However, it would appear that these effects are not robust and may be contingent on more complex semantic or emotional context.

## Supporting information

S1 File Data used in the present study. (XLSX)
